# Lactase Persistence-Associated rs4988235 Polymorphism: A Novel Genetic Link to Cardiovascular Risk via Modulation of ApoB100 and ApoAI

**DOI:** 10.3390/nu17172741

**Published:** 2025-08-24

**Authors:** Nihad Kharrat Helu, Habib Al Ashkar, Nora Kovacs, Roza Adany, Peter Piko

**Affiliations:** 1 Doctoral School of Health Sciences, University of Debrecen, 4032 Debrecen, Hungary; nihad.kharrat.helu@mailbox.unideb.hu (N.K.H.); habib.al.ashkar@med.unideb.hu (H.A.A.); 2 HUN-REN-UD Public Health Research Group, Department of Public Health and Epidemiology, Faculty of Medicine, University of Debrecen, 4032 Debrecen, Hungaryadany.roza@med.unideb.hu (R.A.); 3 Department of Public Health and Epidemiology, Faculty of Medicine, University of Debrecen, 4032 Debrecen, Hungary; 4 National Laboratory for Health Security, Center for Epidemiology and Surveillance, Semmelweis University, 1089 Budapest, Hungary; 5 Institute of Preventive Medicine and Public Health, Semmelweis University, 1089 Budapest, Hungary

**Keywords:** lactase persistence, polymorphism, SNP, Roma, Hungarian, cardiovascular risk, SCORE, SCORE2, Framingham Risk Score, ACC/AHA Pooled Cohort Equations

## Abstract

**Background/Objectives**: As part of the human adaptation to dairy consumption, the presence of the rs4988235-T variant in the *MCM6* gene primarily determines lactase persistence in adult European populations, increasing the expression of the lactase-encoding LCT gene. Carriers of the C/C variant are lactose intolerant, while carriers of the T/T or T/C variant have persistent lactase enzyme activity and are able to digest lactose in adulthood. While the association between lactose intolerance and increased cardiovascular risk (CVR) is well-known, the underlying causes have only been partly explored. The present study aimed to investigate the association of rs4988235 polymorphism with significant lipids affecting cardiovascular health and estimated CVR. **Methods**: The rs4988235 polymorphism was genotyped in 397 subjects from the general Hungarian population and 368 individuals from the Roma population. To characterize the overall lipid profile, total cholesterol (TC), low-density lipoprotein cholesterol (LDL-C), triglycerides (TG), high density lipoprotein cholesterol (HDL-C), apolipoprotein AI (ApoAI), and apolipoprotein B100 (ApoB100) levels were measured, and their ratios (TG/HDL-C, LDL-C/HDL-C, and ApoB100/ApoAI) were calculated. Cardiovascular risk was estimated using the Framingham Risk Score (FRS), Pooled Cohort Equations (PCE), Revised Pooled Cohort Equations (RPCE), and the Systematic Coronary Risk Evaluations (SCORE and SCORE2) algorithms. Adjusted linear and logistic regression analyses were performed, with *p* < 0.05 considered significant. **Results**: The Roma population had a significantly higher prevalence of the C/C genotype than the general population (65.5% vs. 40.3%, respectively). The results of the adjusted linear regression analysis showed a significant association between the C/C genotype and higher LDL-C level (B = 0.126, *p* = 0.047) and ApoB100 level (B = 0.046, *p* = 0.013), as well as a higher LDL-C/HDL-C ratio (B = 0.174, *p* = 0.021) and a higher ApoB100/ApoAI ratio (B = 0.045, *p* = 0.002), as well as a lower HDL-C level (B = −0.041, *p* = 0.049). The C/C genotype was also significantly associated with an increased cardiovascular risk (CVR) as estimated by the SCORE (B = 0.235, *p* = 0.034), SCORE2 (B = 0.414, *p* = 0.009), PCE (B = 0.536, *p* = 0.008), and RPCE (B = 0.289, *p* = 0.045) but not the FRS. After adjusting the statistical model further for ApoAI and ApoB100 levels, the significant correlation with the risk estimation algorithms disappeared (SCORE: *p* = 0.099; SCORE2: *p* = 0.283; PCE: *p* = 0.255; and RPCE: *p* = 0.370). **Conclusions**: Our results suggest that the C/C genotype of rs4988235 is associated with significantly higher ApoB100 and lower ApoAI levels and consequently higher ApoB100/ApoAI ratios, potentially contributing to an increased risk of cardiovascular disease. The results of the statistical analyses suggest that the association between lactose intolerant genotype and cardiovascular risk may be mediated indirectly via modification of the apolipoprotein profile.

## 1. Introduction

Milk and dairy products are a standard component of the traditional Western diet and are recommended in most dietary guidelines worldwide. Lactase persistence (LP) is a trait in which lactose can be digested throughout adulthood, while lactase non-persistence (LNP) can cause lactose intolerance and influence dairy consumption. A single nucleotide polymorphism (SNP ID: rs4988235) is often used as a predictor for dairy intake, since it is responsible for LP in the Caucasian population [1]. On chromosome 2, the rs4988235 SNP in the *LCT* gene (encoding lactase–phlorizin hydrolase) determines LNP status, resulting in higher levels of lactase–phlorizin hydrolase in T/T homozygotes compared to individuals with the C/C genotype [2,3]. In heterozygotes (C/T), lactase levels are intermediate based on both enzyme activity [4] and mRNA levels, but they still produce enough lactase to remain asymptomatic.

The prevalence of LNP in different populations varies widely, from less than 5% in northern Europe to almost 100% in south-east Asia [5]. The T allele is common in northern Europeans, allowing them to digest lactose into adulthood and reflecting a long history of dairy farming, whereas the C allele predominates in regions with high rates of lactose intolerance. Consequently, dairy consumption is traditionally lower in many Asian cultures. Notably, among the Hungarian general population the prevalence of LNP has been estimated at 37%, compared to 56% among Hungarian Roma [6]. The observed ethnic and socioeconomic patterns suggest that the consumption of dairy products and their associated health effects are influenced by various factors, including genetics, culture, environment, and lifestyle.

Recommendations for dairy product consumption are based partly on culture and availability but primarily on meeting nutrient needs. Dairy products are rich in essential micronutrients—including calcium, riboflavin, vitamin B12, and vitamin D—and in most countries two or three servings per day are recommended [7]. The health benefits of milk consumption are positive [8], reducing the risk of osteoporosis [9], type 2 diabetes [10,11], and metabolic syndrome [12]; however, studies examining the relationship between milk consumption and cardiovascular risk have produced inconsistent results. Some studies describe milk consumption as protective against cardiovascular disease [13], while others describe it as a risk factor [14], or find no association [15].

Recent research indicates that the cardiovascular effects of dairy consumption may depend on the type of product consumed. Fermented dairy products, such as yogurt and cheese, contain bioactive peptides and probiotics that are thought to have beneficial effects on lipid profiles and inflammatory markers [14,16,17]. In contrast, unfermented dairy products may lack these protective components, which could explain their association with an increased cardiovascular risk in certain populations. In addition, integrating direct dietary intake data (e.g., through food frequency questionnaires) with genetic markers could clarify whether observed alterations in lipid profiles among LNP individuals are driven solely by reduced dairy intake or by compensatory dietary behaviours.

The positive effects of milk consumption are presumably not related to its lipid composition. Salehi et al. [13] found that consuming both high-fat and low-fat dairy products was associated with beneficial cardiovascular effects. Higher consumption of low-fat dairy products has been associated with a lower Framingham Risk Score (FRS), indicating a reduced 10-year risk of cardiovascular disease in women. Furthermore, yoghurt and cheese consumption have been linked to improved lipid profiles, including lower triglyceride and low-density lipoprotein cholesterol (LDL-C) levels, which are key determinants of cardiovascular risk.

Based on the current literature, the rs4988235 C/C genotype has also been associated with several adverse health outcomes, such as lower bone density [9,18], poorer glucose metabolism [19], and impaired insulin metabolism [20], as well as a higher risk of type 2 diabetes [18], obesity [21], and neural tube defects [22]. Intriguingly, however, it is also linked with a lower risk of Crohn’s disease [23].

Given that cardiovascular disease (CVD) is closely linked to an individual’s lipid profile, which encompasses total cholesterol (TC), LDL-C, high-density lipoprotein cholesterol (HDL-C), and triglycerides (TG), it is crucial to determine whether the metabolic consequences of LNP, mediated by reduced dairy consumption, contribute to an increased risk of CVD.

Cardiovascular risk assessment models such as the Framingham Risk Score (FRS), the Systematic Coronary Risk Evaluation (SCORE) and the American College of Cardiology/American Heart Association (ACC/AHA) Pooled Cohort Equations (PCE) estimate the likelihood of developing cardiovascular disease. These models take into account various factors including age, gender, blood pressure, smoking status, diabetes, and lipid profile. Lipid levels, particularly TC and HDL-C, play a crucial role in these calculations, as high TC and low HDL-C levels significantly increase cardiovascular risk [24]. Furthermore, emerging evidence suggests that more detailed lipid metrics, such as LDL particle size and triglyceride levels, could enhance the accuracy of risk prediction [25].

This study aimed to answer the following questions: Is the rs4988235 C/C genotype associated with cardiovascular risk lipids, namely TC, LDL-C, TG, HDL-C, apolipoprotein AI (ApoAI), and apolipoprotein B100 (ApoB100) levels, as well as their ratios (TG/HDL-C and ApoB100/ApoAI)? If so, can the influence of this on cardiovascular risk be estimated using the FRS, PCE and Revised Pooled Cohort Equations (RPCE), SCORE, and Systematic Coronary Risk Evaluation 2 (SCORE2) algorithms?

Importantly, this study introduces a novel perspective by linking the rs4988235 polymorphism not only to lactose metabolism but also to cardiovascular risk via modulation of the lipid profile (specifically, apolipoprotein levels and the ApoB100/ApoAI ratio). This genetic–lipid–CVR axis represents an unexplored pathway with potential clinical relevance.

## 2. Materials and Methods

### 2.1. Study Design and Populations

A complete and detailed description of the study design and the data collection was presented in our previous papers [26,27]. Briefly, to understand the background of the poor health status of the Roma population compared with that of the Hungarian population, especially the high incidence of non-communicable diseases, a health survey was designed and implemented to create a complex database for comparative and association statistical analysis. The survey received ethical approval in 2017 (Reference No.: 61327-3/2017/EKU).

The Roma sample was drawn from 25 randomly selected segregated Roma colonies with a population of more than 100 people. From each colony, 20 households and one person from each household were randomly selected. For the Hungarian general (HG) sample, 25 randomly selected individuals from each of the 20 randomly selected general practices of the General Practitioners’ Morbidity Sentinel Stations Program (GPMSSP) were invited to participate in the study.

The study consisted of three main components (a questionnaire-based survey, physical and laboratory examinations) in the Hungarian general and Roma adult populations aged 20–64 years. A total of 832 participants were included in the survey, 417 from the HG population (232 women and 185 men) and 415 from the Roma population (307 women and 108 men).

A three-pillar health survey was conducted, consisting of questionnaire-based interviews, physical examinations and laboratory analyses.

Questionnaire-based interviews, using the European Health Interview Survey (EHIS) Wave 2 instrument, were extended with validated modules on health determinants and socio-economic status.

Physical examinations, following the European Health Examination Survey protocol, including anthropometric and blood pressure measurements, visual acuity, and cardiovascular fitness.

Laboratory analyses were performed on fasting blood samples to assess lipid profiles, glucose, insulin, creatinine, uric acid, liver enzymes, and apolipoproteins. Genomic DNA was extracted for genotyping special sets of polymorphisms using the MassARRAY platform.

### 2.2. Questionnaire-Based Interviews

The core instrument for the questionnaire-based interviews was the European Health Interview Survey (EHIS) Wave 2, adapted for Hungary in 2014. This covered the following four areas: (a) health status (self-perceived health, chronic conditions, limitations, and mental health); (b) healthcare use (hospitalisations, consultations, preventive services, medications, and unmet needs); (c) health determinants (smoking, alcohol consumption, physical activity, and dietary habits); and (d) socio-economic variables (e.g., sex, age, education, income, employment, and living conditions).

In addition to the standard EHIS modules, the questionnaire was extended with validated items from previous national surveys to capture further health-related behaviours and contextual factors. Interviews were conducted face-to-face by trained Roma university students under the supervision of public health coordinators, ensuring cultural sensitivity and high data quality. All responses were recorded using structured forms, and data validation followed Eurostat’s EHIS 2 protocol, including skip logic, range checks, and consistency rules.

### 2.3. Physical Examinations

Standardised anthropometric and blood pressure measurements were performed according to the European Health Examination Survey protocol. Height, weight, and waist circumference were recorded, and body mass index (BMI) was calculated. In addition to these core assessments, physical examinations included an evaluation of visual acuity and cardiovascular fitness, performed by trained medical staff. The cardiovascular fitness assessment was based on routine clinical evaluation methods adapted for field conditions. All measurements were carried out in general practitioners’ offices by qualified personnel, ensuring consistency across study sites.

### 2.4. Laboratory Analyses

Lipid levels were determined from fresh serum. TC and TG levels were measured using an enzymatic colorimetric method (GPO-PAP, Modular P-800 Analyzer; Roche/Hitachi, Basel, Switzerland). HDL-C and LDL-C levels were measured using a homogeneous enzymatic colorimetric assay (HDL-C plus 3rd generation, LDL-C plus 2nd generation, Roche, Basel, Switzerland). Apolipoprotein (ApoAI and ApoB100) levels were determined by immunoturbidimetric assay Tina-quant apolipoprotein A-1 (Version 2; Roche, Basel, Switzerland), and Tina-quant apolipoprotein B (Version 2; Roche, Basel, Switzerland).

Creatinine, glucose, insulin, uric acid, and gamma-glutamyl transferase (GGT) levels were measured from fasting serum samples using standardized enzymatic and colorimetric methods on the Modular P-800 Analyzer (Roche Diagnostics, Basel, Switzerland). All assays were performed according to the manufacturer’s protocols, with internal quality control procedures applied.

Genomic DNA was isolated using the MagNA Pure LC system and MagNA Pure LC DNA Isolation Kit-Large (Roche Diagnostics, Basel, Switzerland), following the manufacturer’s instructions. Genotyping of cholesteryl ester transfer protein (*CETP*) gene polymorphisms rs1532624 and rs5882 was conducted by the Mutation Analysis Core Facility (MAF) at Karolinska University Hospital, Sweden, using the MassARRAY platform (Sequenom Inc., San Diego, CA, USA) and iPLEX Gold chemistry. Validation, concordance analysis, and quality control were performed in accordance with MAF protocols.

### 2.5. Scores Used to Estimate Cardiovascular Risk in Study Populations

In this study, we used the most widely used risk assessment models (FRS, PCE, RPCE, and SCOREs) in Europe to estimate cardiovascular risk. All algorithms are sex-specific and estimate the likelihood of a cardiovascular event occurring within 10 years.

Data from the Framingham Heart Study [28] were used to develop the first version of the FRS which was designed to estimate the 10-year risk of developing coronary heart disease (CHD) [29]. It was later revised and optimised for use in estimating the risk of developing CVD [30]. Both versions consider age, sex, TC, HDL-C levels, systolic blood pressure, antihypertensive treatment, and current smoking status. In addition to these factors, the FRS–CVD algorithm also considers the presence of diabetes when calculating risk. As the risk estimated by the FRS algorithm is considered valid for people aged 30–79 years, FRS calculations were performed for people aged 30–64 years in the present analysis.

The PCE [31] and the RPCE [32] are cardiovascular risk assessment models developed by the American College of Cardiology and the American Heart Association. They estimate an individual’s 10-year risk of developing atherosclerotic cardiovascular disease (ASCVD), including coronary heart disease and stroke.

The PCE (2013) was introduced to enhance the predictive capabilities of traditional models, such as the Framingham Risk Score. It considers various factors, such as age, gender, race, cholesterol levels, blood pressure, diabetes status, and smoking habits, in order to evaluate the risk of ASCVD. The RPCE (2018) is a revised version of the PCE, designed to address concerns about the original model overestimating risk. It uses updated statistical methods and modern cohort data to provide more accurate risk predictions. The PCE and RPCE analyses were performed on participants aged between 40 and 64 years in the study populations.

The SCORE risk estimate, which is based on an algorithm recommended by the European Society of Cardiology in its 2007 guidelines for the prevention of cardiovascular disease in clinical practice, was also calculated. Additionally, the SCORE2 algorithm, which is designed to predict the 10-year risk of first-onset cardiovascular disease in the European population, was calculated. It was calibrated and validated to improve the identification of individuals at higher risk of developing fatal and non-fatal cardiovascular disease across Europe. In the present study, we used the SCORE and SCORE2 algorithms for high-risk countries (due to the Hungarian origin of the samples). The risk factors included in these algorithms are sex, age, smoking status, systolic blood pressure, TC, and HDL-C. All SCORE analyses were performed on participants aged between 40 and 64 years in the study populations.

A more detailed explanation of the cardiovascular risk models used in this study can be found in our previous publication [33].

### 2.6. Statistical Analyses

Categorical variables were compared using the χ2 test, while comparisons between two subgroups were made using the Mann–Whitney U-test. The Kolmogorov–Smirnov test was used to determine whether the quantitative variables were normally distributed and, if necessary, Templeton’s two-step method [34] was used to transform the non-normally distributed variables into normal ones. The Jonckheere–Terpstra trend test [35] was used to identify the statistically significant trends between the ordinal independent variables and the continuous or ordinal dependent variables. Correlations between continuous variables were assessed via multiple linear and logistic regression analyses.

All statistical models used in the basic analyses were adjusted for the following factors: ethnicity, sex, age, body mass index (BMI), waist circumference, education level, lipid-lowering therapy, antidiabetic treatment, antihypertensive treatment, current smoking status, lactose-free diet, serum creatinine, systolic and diastolic blood pressure, glucose, insulin, uric acid, GGT, and *CETP* gene polymorphisms (rs1532624 and rs5882). To investigate potential mediating effects, the models were additionally adjusted for ApoB100 and ApoAI levels.

To identify mediating variables, Sobel–Goodman mediation analysis was performed to estimate the indirect effect of the predictor on the outcome via the proposed mediator. This method enabled us to evaluate the magnitude and statistical significance of the mediation effect, thereby clarifying the relationship between the independent and dependent variables.

In the analysis of the individual effects of lipids and their ratios as outcome variables, corrections were made for all additional lipids that were statistically independent (LDL, HDL-C, and TG as well as ApoB100, ApoAI, and TG). No correction was made for additional lipids in the analysis of total cholesterol.

## 3. Results

### 3.1. Basic Characteristics and Lipid Profile

Homozygous carriers of the C allele are significantly more frequently found among Roma individuals (60.10% vs. 34.89%, *p* < 0.001), more likely to be female (70.32% vs. 57.42%, *p* < 0.001), current smokers (56.36% vs. 40.38%, *p* < 0.001), to have lower levels of education (*p* < 0.001), and a significantly different genotype frequency of the rs5882 (*p* = 0.046). Additionally, laboratory tests show significantly lower levels of uric acid (C/C = 284.32 µmol/L vs. T/T or C/T = 263.02 µmol/L) and creatinine (C/C = 65.88 µmol/L vs. T/T or C/T = 62.39 µmol/L) levels among homozygotes for the C allele. See Table 1 for further details.

Stratified analyses by ethnicity showed that, within the Hungarian general population (Supplementary Table S1), C-allele homozygotes had significantly lower fasting glucose (5.09 vs. 5.36 mmol/L, *p* = 0.032), uric acid (276.68 vs. 294.97 µmol/L, *p* = 0.016), and creatinine (63.97 vs. 67.00 µmol/L, *p* = 0.021) levels than T-allele carriers, with no significant differences in smoking, education, or treatment variables. In the Roma population (Supplementary Table S2), C-allele homozygotes were more likely to be current smokers (70.54% vs. 57.48%, *p* = 0.012), but no significant differences emerged for lipid-lowering, antihypertensive, or antidiabetic treatment, anthropometric or most laboratory measures.

Full unadjusted descriptive comparisons between genotype groups are presented in Table 1 (overall sample) and in Supplementary Tables S1 (Hungarian general population) and S2 (Roma population).

### 3.2. Association of rs4988235—C/C Genotype with Lipid Parameters

Among the tested lipid parameters, carriers of the C/C genotype had significantly lower ApoAI levels (C/C = 1.48 vs. T/T or C/T = 1.52, *p* = 0.024) and significantly higher ApoB100 levels (C/C = 1.08 vs. T/T or C/T = 1.04, *p* = 0.036) and ApoB100/ApoAI ratios (C/C = 0.75 vs. T/T or C/T = 0.71, *p* = 0.008) compared to carriers of the T/T or T/C genotypes. There were no significant differences in TC, LDL-C, TG, HDL-C, and LDL-C /HDL-C and TG/HDL-C ratios between the two groups. See more details in Table 2.

The C/C genotype was significantly associated with increased LDL-C level (B = 0.126, 95%CI: 0.001–0.251; *p* = 0.047), ApoB100 level (B = 0.046, 95% CI: 0.011–0.080, *p* = 0.010), and LDL-C/HDL-C ratio (B = 0.174, 95%CI: 0.026–0.322; *p* = 0.021) and ApoB100/ApoAI ratio (B = 0.045, 95% CI: 0.016–0.074, *p* = 0.002), as well as a reduced HDL-C level (B = −0.041, 95%CI: −0.081–−0.000; *p* = 0.049).

**Figure 1:**
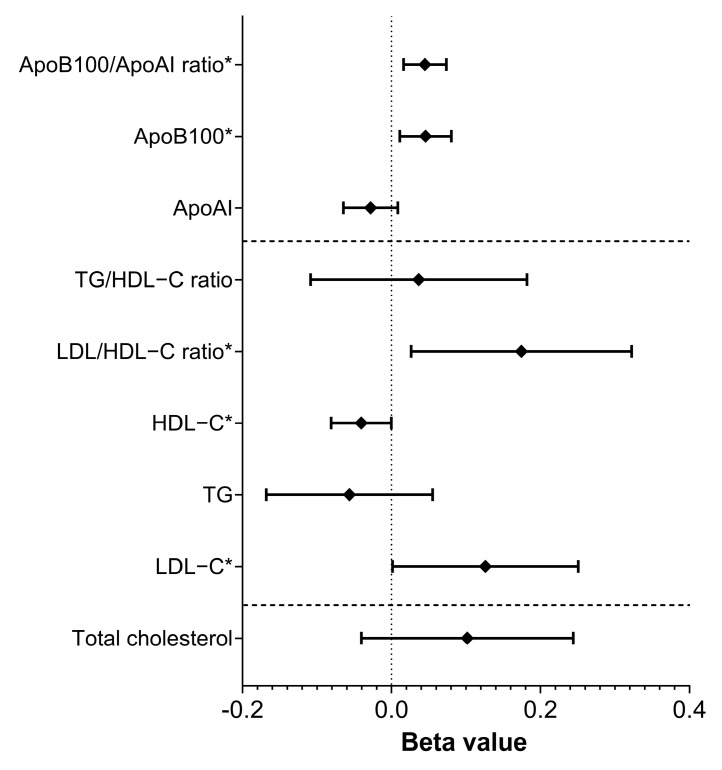
Association of rs4988235—C/C genotype with lipid parameters as continuous variables. Statistically significant results are highlighted in bold. *: *p* < 0.05; LDL-C: low-density lipoprotein cholesterol; TG: triglycerides; HDL-C: high density lipoprotein cholesterol; ApoAI: apolipoprotein AI; ApoB100: apolipoprotein B100.

**Figure 2:**
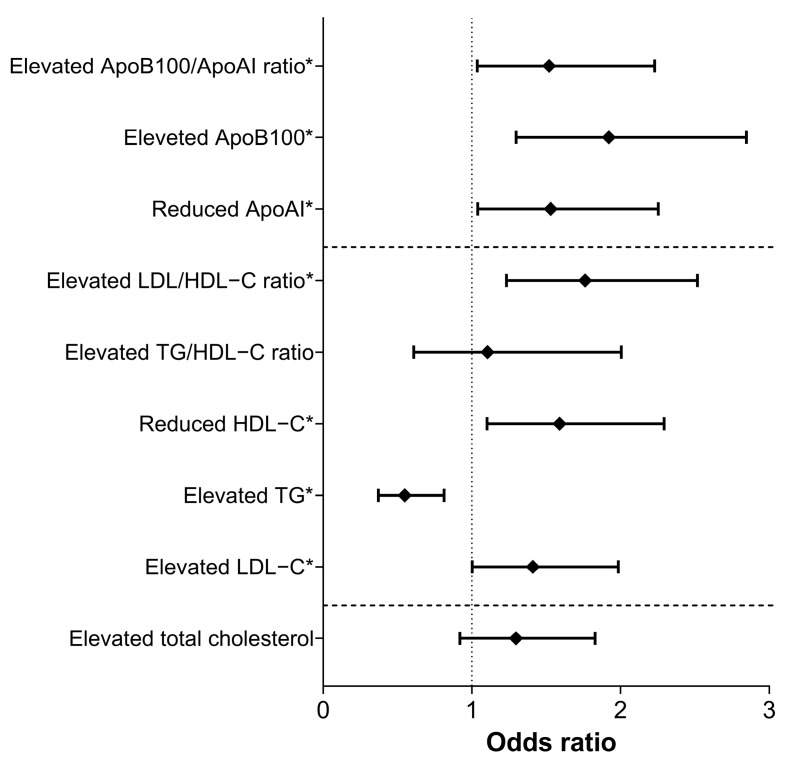
Association of rs4988235—C/C genotype with lipid parameters as categorical variables. Statistically significant results are highlighted in bold. *: *p* < 0.05; LDL-C: low-density lipoprotein cholesterol; TG: triglycerides; HDL-C: high density lipoprotein cholesterol; ApoAI: apolipoprotein AI; ApoB100: apolipoprotein B100.

Furthermore, rs4988235—C/C genotype was significantly associated with a higher risk of reduced HDL-C (odds ratio [OR] = 1.589, 95% CI: 1.102–2.384, *p* = 0.013) level and ApoAI level (OR = 1.530, 95% CI: 1.039–2.254, *p* = 0.031), increased LDL-C level (OR = 1.411, 95% CI: 1.002–1.501, *p* = 0.049), and ApoB100 level (OR = 1.922, 95% CI: 1.298–2.846, *p* = 0.001), and increased LDL/TG ratio (OR = 1.744, 95% CI: 1.233–2.518, *p* = 0.002), and a lower risk of increased (≥1.7 mmol/L) TG level (OR = 0.549, 95% CI: 0.371–0.813, *p* = 0.003). See Figure 1 and Figure 2 for further details.

### 3.3. Association of rs4988235–C/C Genotype with Estimated Cardiovascular Risk

Of the six risk estimation models tested, four showed a significantly higher cardiovascular risk among C/C genotype carriers compared to T/T or T/C carriers: SCORE (2.95 vs. 2.23, *p* = 0.039), SCORE2 (7.09 vs. 5.49, *p* = 0.001), PCE (6.76 vs. 5.35, *p* = 0.005), and RPCE (5.56 vs. 4.15, *p* = 0.017). No significant difference was seen for the FRS risk estimation models. See Table 3 for more details.

**Figure 3:**
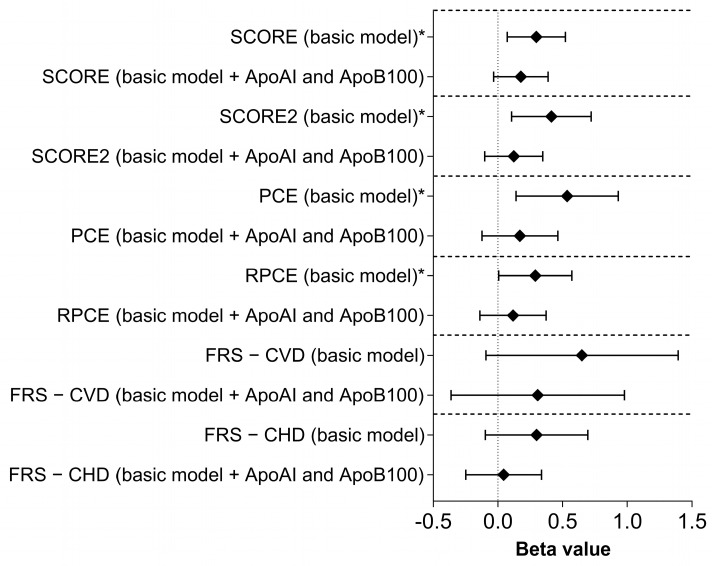
The association of the rs4988235-C/C genotype with the Systematic Coronary Risk Evaluation (SCORE and SCORE2), Pooled Cohort Equations (PCE), Revised Pooled Cohort Equations (RPCE), and the Framingham Risk Score for cardiovascular disease (FRS—CVD) and coronary heart disease (FRS—CHD) is based on basic model (adjusted for ethnicity, sex, age, education, waist circumference, lipid-lowering treatment, antidiabetic treatment, antihypertensive treatment, current smoking status, lactose-free diet, creatinine level, insulin level, uric acid level, GGT level, and TG level, as well as rs1532624 and rs5882 SNPs in the *CETP* gene). Basic model was further adjusted for ApoB100 and ApoAI levels. * *p* < 0.05.

The rs4988235 C/C genotype was significantly associated with an increased cardiovascular risk, as estimated by the SCORE (B = 0.235, 95%CI: 0.018–0.451; *p* = 0.034), SCORE2 (B = 0.414, 95%CI: 0.106–0.721; *p* = 0.009), PCE (B = 0.536, 95%CI: 0.140–0.931; *p* = 0.008), and RPCE (B = 0.289, 95%CI: 0.006–0.573; *p* = 0.045). However, no significant associations were observed with the FRS_CVD_ (B = 0.470, 95%CI: −0.090–1.029; *p* = 0.100) or FRS_CHD_ (B = 0.223, 95%CI: −0.151–0.597, *p* = 0.242) (basic model). After further adjusting the statistical model for the ApoB100 and ApoAI levels, the significant correlation between the rs4988235 C/C genotype and SCORE (B = 0.117, 95% CI: −0.082–0.317; *p* = 0.099), SCORE2 (B = 0.123, 95%CI: −0.102–0.347; *p* = 0.283), PCE (B = 0.170, 95%CI: −0.123–0.464; *p* = 0.255), and RPCE (B = 0.117, 95%CI: −0.139–0.373; *p* = 0.370) disappeared. See Figure 3 for further details.

**Figure 4:**
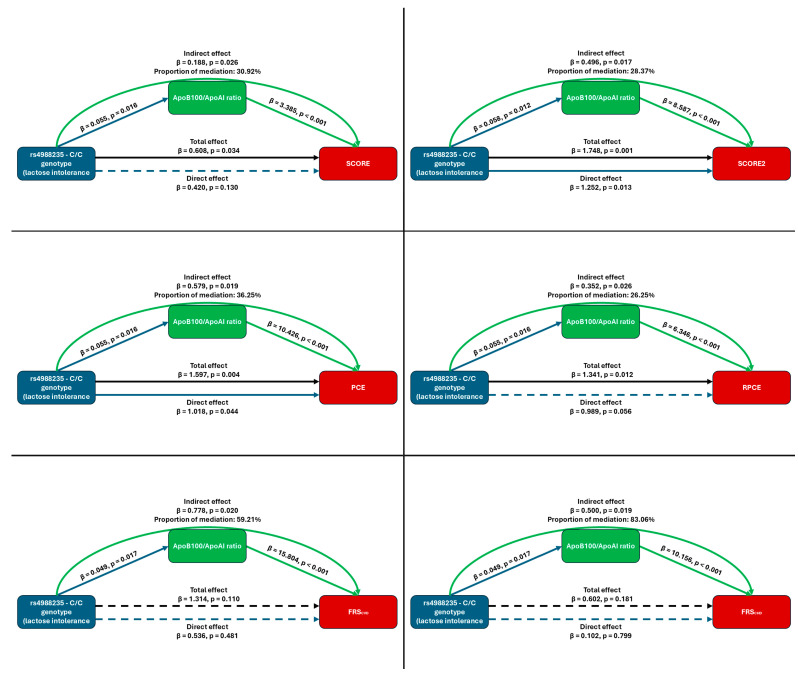
Path diagram illustrating the Sobel–Goodman mediation analysis for the Systematic Coronary Risk Evaluation (SCORE and SCORE2), Pooled Cohort Equations (PCE), Revised Pooled Cohort Equations (RPCE), and the Framingham Risk Score for cardiovascular disease (FRS_CVD_) and coronary heart disease (FRS_CHD_). Solid arrows represent statistically significant paths (*p* < 0.05), while dashed arrows indicate non-significant associations (*p* ≥ 0.05). Path coefficients (β) and corresponding *p*-values are shown alongside each arrow. The indirect effect, representing the portion of the genotype–risk score association mediated through the ApoB100/ApoAI ratio, is presented with its β estimate, *p*-value, and percentage of the total effect. In the figure, blue indicates genotype, green indicates ApoB100/ApoAI, and black indicates the total effect in the process.

Sobel–Goodman mediation analyses revealed that the rs4988235 polymorphism significantly influences multiple cardiovascular risk scores through the ApoB/ApoAI ratio as a mediator. For SCORE2, the total effect was β = 1.748 (95% CI: 0.690–2.805; *p* = 0.001), of which 28.4% was mediated via ApoB/ApoAI (indirect effect = 0.496; *p* = 0.017). Similarly, for PCE a total effect of β = 1.597 (95% CI: 0.507–2.687; *p* = 0.004), with 36.2% mediation (indirect effect = 0.579; *p* = 0.019). For RPCE, the indirect effect was β = 0.352 (*p* = 0.026), accounting for 26.3% of the total association. While direct effects remained significant for most risk estimation algorithms, mediation strength varied: for SCORE, the direct effect become non-significant when the mediator was included (β = 0.420; *p* = 0.130), suggesting partial mediation. In contrast, both FRS_CVD_ and FRS_CHD_ models exhibited strong indirect effects (β = 0.778 and β = 0.500, respectively; both *p* < 0.05), despite non-significant direct paths. These findings consistently highlight the ApoB/ApoAI ratio as a significant intermediary. For more details see Figure 4.

## 4. Discussion

Our primary objective was to investigate the association between the rs4988235 polymorphism—a well-known genetic variant involved in lactase persistence—and cardiovascular lipid profiles. The T allele of this polymorphism is traditionally associated with lactase persistence in adults, whereas individuals with the C/C genotype tend to experience lactase non-persistence. While lactase persistence is typically considered from a nutritional standpoint, our findings suggest that the genetic background for lactose digestion may also affect serum lipid levels and cardiovascular risk markers.

After adjusting for confounding factors, we found that the C/C genotype was significantly associated with elevated LDL-C levels, reduced HDL-C levels, and a higher LDL/HDL ratio. Interestingly, this genotype also correlated with a lower risk of elevated triglyceride (TG) levels. The inverse association between the C/C genotype and TG concentrations may be explained by enhanced clearance of triglyceride-rich lipoproteins or by differences in hepatic very-low-density lipoprotein (VLDL) secretion rates [36]. Furthermore, genetic variation at the LCT/MCM6 locus could indirectly affect lipid metabolism through mechanisms such as modulation of gut microbiota composition, bile acid signalling, or intestinal lipid absorption efficiency [37,38]. In addition, population-specific dietary habits associated with lactase persistence genotypes—particularly variations in habitual dairy fat intake—may further influence triglyceride homeostasis and contribute to the protective lipid profile observed in individuals with the C/C genotype [39].

In line with this, significantly higher ApoB100 (the main apolipoprotein of LDL) levels and ApoB100/ApoAI ratios were observed. One possible explanation for these associations is related to dietary patterns. Individuals with lactase non-persistence (i.e., those with the C/C genotype) may naturally avoid dairy products due to discomfort experiencing after consuming food containing lactose [40,41]. Dairy products, especially fermented ones, have been associated with beneficial effects on lipid metabolism owing to their bioactive compounds, micronutrients, and even probiotic content [42]. Therefore, reducing dairy intake among individuals with the C/C genotype could lead to an increase in consumption of other food groups that may offer less cardiovascular protection [43]. For example, replacing dairy with foods high in saturated fats or refined carbohydrates could result in higher LDL cholesterol levels, increased ApoB levels, and less favourable lipid ratios [43,44]. Conversely, lactase-persistent individuals may benefit from the potential protective effects of dairy components, which could help maintain a balanced lipid profile [45].

Beyond dietary modifications, genetic pleiotropy may partly explain these results. Although rs4988235 has primarily been studied for its role in regulating lactase enzyme activity, it is possible that this variant or other genetic loci in linkage disequilibrium with it, influences lipid metabolism [1]. For instance, regulatory elements around the MCM6 gene could affect neighbouring genes that are directly involved in modulating cholesterol synthesis, transport, or clearance [46]. This pleiotropic effect may predispose individuals with the C/C genotype to an atherogenic lipid profile, independently of their dairy consumption. Therefore, the elevated LDL levels, unfavourable LDL/HDL ratios, and increased ApoB100/ApoAI ratios observed in the study could be manifestations of the broader metabolic impact of this genomic region.

The gut microbiota may also contribute. Individuals who are lactase non-persistent typically experience undigested lactose reaching the colon, where it is fermented by bacteria [47,48]. This fermentation process can alter the composition and function of the gut microbiome, potentially leading to changes in systemic metabolism. The microbiota plays a central role in modulating lipid absorption, bile acid metabolism, and inflammation, all of which are key factors in determining serum lipid levels and cardiovascular risk [49,50]. An imbalanced microbiome could contribute to subtle shifts in lipid parameters, which may partly explain why individuals with the C/C genotype exhibit markers of dyslipidaemia [51].

Changes in lipid metabolism are not isolated events; they are intertwined with systemic metabolic processes such as insulin sensitivity [52] and inflammation [53]. In lactase-persistent individuals, milk consumption can modify the effects of insulin, which in turn can influence lipoprotein production patterns [19]. Reduced insulin sensitivity, for example, has been linked to increased levels of small, dense LDL particles and decreased HDL levels. Therefore, the association between rs4988235 and LDL and HDL levels may be partly mediated by these interrelated metabolic factors.

Other possible influencing factors may include bioactive components found in dairy products, such as calcium and milk proteins, which can influence lipoprotein metabolism and thus ApoB levels [54,55].

Calcium can bind to fatty acids and cholesterol in the small intestine, thereby inhibiting their absorption [56,57]. These less soluble complexes are then excreted by the body, reducing the amount of cholesterol that is absorbed and subsequently affecting lipoprotein synthesis in the liver.

The digestion of milk proteins, such as casein and whey, releases bioactive peptides that can improve insulin sensitivity [58,59]. Optimal insulin action promotes normal lipid synthesis and distribution, thereby contributing to a favourable lipid profile. Population genetics adds another layer of complexity. Our study found that the Roma population had a significantly higher prevalence of the C/C genotype compared to the general Hungarian population. This genotype distribution aligns with previous publications [60,61], and notably, the Roma population also exhibits a higher cardiovascular risk overall compared to the Hungarian general population, as reported in prior comparative assessments [33]. This difference may reflect distinct evolutionary histories and dietary adaptations; however, it also serves as a reminder that genetic predispositions can interact with cultural, socioeconomic, and environmental factors. In populations where alternative dietary habits and limited access to healthcare and/or other lifestyle-related cardiovascular risk factors are prevalent, the genetic influence may be amplified. In this context, the association between the C/C genotype and an atherogenic lipid profile may partially reflect the underlying health disparities and lifestyle factors prevalent within the population.

Interestingly, after adjusting for ApoB100 and ApoAI levels, the associations with the cardiovascular risk scores estimated using SCORE, SCORE2, PCE, and RPCE were no longer statistically significant. These results reinforce the notion that the ApoB/ApoA-I ratio consistently mediates the relationship between the rs4988235 genotype and cardiovascular risk. Mediation effects were significant across all models, with indirect contributions ranging from 26.3% to 36.2%. In models such as FRS_CVD_ and FRS_CHD_, the presence of non-significant direct effects alongside strong indirect effects suggests the operation of a complete mediation mechanism. This points to a lipid-regulated genetic pathway by which rs4988235 may influence cardiometabolic risk.

These findings imply that the adverse impact of the C/C genotype on cardiovascular risk is mediated by these apolipoprotein markers. In other words, the genetic predisposition primarily leads to changes in the ApoB100/ApoAI ratio, a well-established marker of atherogenic risk, and it is these changes that influence the estimation of overall cardiovascular risk [62,63,64]. These findings suggest that interventions targeting lipid management, particularly the balance between ApoB- and ApoAI-containing lipoproteins, could reduce some of the cardiovascular risks associated with the C/C genotype.

Zhang et al.’s study [16] examined the relationship between milk consumption, the rs4988235 variant, and CVD risk in a Swedish cohort of 20,499 individuals who were monitored for an average of 21 years. The findings showed that higher intake of non-fermented milk was linked to increased CHD and CVD mortality, whereas higher consumption of fermented milk was associated with a lower risk of CVD and mortality. Additionally, the rs4988235 genotype, which is associated with higher milk intake (particularly non-fermented milk), was positively linked to an increased risk of CHD and CVD (CT/TT vs. CC: CHD risk higher by 27%; CVD risk higher by 22%). The impact of the genotype on CVD mortality was stronger in individuals with higher milk intake, suggesting a gene–diet interaction. Furthermore, non-fermented milk intake was associated with higher leptin levels and altered HDL levels, which may contribute to cardiovascular risk. No significant associations were found between fermented milk intake and lipoprotein subfractions. Overall, these findings suggest that the type of milk consumed, and genetic predisposition play a significant role in cardiovascular health.

The Swedish study found an association between lactase persistence (CT/TT genotype) and a higher intake of non-fermented milk, which leads to an increased risk of CVD and CHD, potentially mediated by alterations in HDL cholesterol and leptin levels. By contrast, our study found that the C/C genotype, which is linked to lactose intolerance, was associated with higher LDL-C and ApoB100 levels and unfavourable lipid ratios, ultimately increasing cardiovascular risk as estimated by various models (SCORE, SCORE2, PCE, and RPCE). This apparent contradiction can be resolved by recognizing that the two studies explore different mechanisms of risk.

The Swedish cohort emphasized a gene–diet interaction, where LP individuals consumed more non-fermented milk, which was associated with elevated leptin levels and altered HDL profiles, contributing to increased CVD risk. In contrast, our study focused on genotype-driven metabolic alterations, independent of direct dietary intake, showing that LNP individuals had elevated ApoB100 levels, reduced ApoAI, and unfavourable lipid ratios—factors that strongly predict cardiovascular risk.

Thus, while LP may increase risk via dietary behaviour, LNP may elevate risk via intrinsic metabolic pathways, possibly influenced by genetic pleiotropy, microbiome composition, or compensatory dietary patterns. These findings are complementary, illustrating that both genotypes may elevate cardiovascular risk—albeit through divergent pathways: one behavioural, the other metabolic. This duality highlights the importance of integrating genetic, dietary, and metabolic data when assessing cardiovascular risk.

However, when ApoAI and ApoB100 levels were taken into account alongside genotype in the analyses, the association between genotype and cardiovascular risk disappeared. This suggests that these biomarkers mediate the relationship between genotype and risk. The ApoB100/ApoAI ratio is widely recognized as an integrated indicator of atherogenic risk, capturing both the burden of cholesterol-carrying particles and the capacity for reverse cholesterol transport [62,64]. ApoB100 is the principal apolipoprotein of all atherogenic lipoproteins—including LDL, VLDL, IDL, and Lp(a)—and its plasma concentration directly reflects the total number of these particles capable of arterial wall infiltration. In contrast, ApoAI is the major structural protein of HDL particles, which mediate reverse cholesterol transport and exert anti-inflammatory and antioxidative effects within the vasculature.

A higher ApoB100/ApoAI ratio therefore indicates a predominance of atherogenic overprotective lipoproteins—a state consistently associated, in large epidemiological studies with increased incidence of myocardial infarction, stroke, and other ASCVD events. Notably, this ratio often demonstrates superior prognostic performance compared to traditional lipid measures such as LDL-C or total cholesterol.

This dual representation of pro- and anti-atherogenic forces explains why the ApoB100/ApoAI ratio functions as a potent mediator between genetic variation at rs4988235 and cardiovascular risk estimates, as observed in our statistical analyses. It reflects not only lipid quantity but also lipoprotein quality and functionality, making it a biologically plausible and clinically relevant mediator in the genotype–CVR pathway.

Considering these results: LP (T/T and T/C genotypes) may increase cardiovascular risk through dietary habits (such as increased consumption of unfermented milk) and changes in lipid metabolism. Conversely, LNP (C/C genotype) may directly impact lipid metabolism, particularly LDL and ApoB levels, thereby contributing to cardiovascular risk. Therefore, understanding the link between rs4988235 and CVD risk requires consideration of both dietary patterns and genetic lipid regulation.

Importantly, our findings also raise the possibility of clinical applications. Given the strong mediating role of the ApoB100/ApoAI ratio and its superior predictive value compared to traditional lipid markers, routine apolipoprotein testing may be warranted in genetically at-risk populations—particularly among individuals with the rs4988235 C/C genotype. Early identification of an unfavourable ApoB100/ApoAI profile could support more personalized cardiovascular prevention strategies, including dietary counselling, lipid-lowering therapy, and closer monitoring. In populations with high prevalence of lactase non-persistence, such as the Roma community, this approach may offer a practical tool for targeted risk reduction.

Our study has both strengths and limitations. First, due to a lack of information regarding gene–gene and gene–environment interactions, epigenetic factors and structural variants were not considered in the analysis. Furthermore, the cross-sectional nature of the study only provides a snapshot in time, preventing any inference of causality between the rs4988235 C/C genotype and cardiovascular risk. The sample size may also be limited or not fully representative, which could affect the generalizability of the findings. Additionally, certain lifestyle or genetic factors not included in the study may influence the observed associations. A major limitation is the lack of dietary intake data, particularly regarding dairy consumption. Given that rs4988235 polymorphism affects lactase persistence, differences in actual lactose intake could influence lipid metabolism and cardiovascular risk. As dietary habits were not actively controlled or analysed, we cannot exclude the possibility that unmeasured nutritional factors also contributed to the observed associations. Finally, as this is a cross-sectional study, it does not allow changes to be tracked over time, which is crucial for understanding the long-term genetic influence on cardiovascular risk. On the other hand, the present study has several strengths. Notably, it is the first to examine the potential impact of the rs4988235 C/C genotype on apolipoprotein profile, namely ApoAI level, ApoB100 level, their ratio, and cardiovascular risk. The study deepens our understanding of genetic predispositions to cardiovascular disease risk, offering a novel perspective.

## 5. Conclusions

In conclusion, this study demonstrates that the rs4988235 C/C genotype, which is associated with lactase non-persistence, is linked to an adverse lipid profile. This includes elevated LDL-C and ApoB levels, unfavorable lipid ratios, and reduced HDL-C. These alterations correlate with an increased risk of cardiovascular disease, as estimated by SCORE, SCORE2, PCE, and RPCE algorithms, particularly in populations with a high prevalence of the C/C genotype, such as the Roma ethnic community. It is notable that the association appears to be mediated through changes in apolipoprotein levels, as its significance diminishes after adjustment. These findings can be applied in clinical practice to improve cardiovascular risk stratification, inform personalized dietary and lifestyle interventions, and direct future interdisciplinary research into the metabolic effects of lactase non-persistence.

## Figures and Tables

**Table 1:** Unadjusted descriptive comparisons between genotype groups. Categorical variables were analyzed using Pearson’s chi-square test, and continuous variables using the Mann–Whitney U test. No covariate adjustment was applied in this table.

		rs4988235—T/T or T/C (Lactose Tolerance)	rs4988235—C/C (Lactose Intolerance)	*p*-Value
		N = 364	N = 401	
		Prevalence in % (95%CI)		
Roma		34.89 (30.13–39.89)	60.10 (55.25–64.81)	<0.001 *
Women		57.42 (52.30–62.42)	70.32 (65.72–74.64)	<0.001 *
Lipid lowering treatment		7.42 (5.06–10.45)	10.72 (7.89–14.03)	0.113
Antihypertensive treatment		28.85 (24.37–33.65)	30.92 (26.55–35.57)	0.531
Antidiabetic treatment		7.42 (5.06–10.45)	9.48 (6.90–12.63)	0.308
Current smoker		40.38 (25.44–45.48)	56.36 (51.47–61.15)	<0.001 *
Lactose-free diet		1.65 (0.69–3.37)	1.50 (0.63–3.06)	0.866
Education	Primary	45.05 (40.00–50.19)	59.10 (54.24–63.83)	<0.001*
	Secondary	42.58 (37.58–47.70)	33.17 (28.569–37.88)	
	College or university	12.36 (9.28–16.04)	7.73 (5.42–10.65)	
rs1532624 in the *CETP* gene	C/C—genotype	30.77 (26.19–35.65)	33.17 (28.69–37.88)	0.633
	A/C—genotype	47.80 (42.71–52.93)	44.39 (39.68–49.28)	
	A/A—genotype	21.43 (17.45–25.86)	22.44 (18.57–26.72)	
rs5882 in the *CETP* gene	G/G—genotype	12.15 (9.09–15.82)	17.21 (13.76–21.13)	0.046 *
	G/A—genotype	48.34 (43.23–53.49)	40.65 (35.92–45.51)	
	A/A—genotype	39.50 (34.57–44.61)	42.14 (37.38–47.02)	
		Average (95%CI)		*p*-value
Age (years)		43.51 (42.25–44.77)	43.53 (42.33–44.74)	0.945
Waist circumference (cm)		95.97 (94.36–97.58)	94.93 (93.35–96.52)	0.341
BMI (kg/m^2^)		27.64 (27.03–28.25)	27.25 (26.62–27.87)	0.292
Systolic blood pressure (mmHg)		125.10 (123.60–126.59)	125.46 (123.75–127.17)	0.672
Diastolic blood pressure (mmHg)		79.09 (78.18–79.99)	79.32 (78.33–80.31)	0.990
Insulin level (mU/L)		16.01 (14.27–17.75)	16.44 (14.41–18.46)	0.402
Fasting glucose (mmol/L)		5.25 (5.05–5.44)	5.12 (4.96–5.27)	0.281
Uric acid (µmol/L)		284.32 (276.67–291.98)	263.02 (255.57–270.47)	<0.001 *
Creatinine (μmol/L)		65.88 (64.43–67.32)	62.39 (60.87–63.90)	<0.001 *
GGT (U/L)		34.27 (31.15–37.39)	36.64 (29.29–43.99)	0.106

*CETP*: Cholesteryl ester transfer protein gene; BMI: body mass index; GGT: gamma-glutamyl transferase; A: adenine; C: cytosine; G: guanine; T: thymine; *: *p* < 0.05.

**Table 2:** Basic lipid profile of study population.

	rs4988235–T/T or T/C (Lactose Tolerance)	rs4988235–C/C (Lactose Intolerance)	*p*-Value
	N = 361	N = 395	
	Average (95%CI)		
Total cholesterol	4.93 (4.82–5.04)	5.00 (4.90–5.11)	0.195
LDL-C (mmol/L)	3.09 (2.99–3.18)	3.20 (3.10–3.29)	0.092
TG (mmol/L)	1.63 (1.52–1.74)	1.56 (1.46–1.66)	0.349
HDL-C (mmol/L)	1.34 (1.30–1.38)	1.30 (1.26–1.33)	0.093
LDL-C/HDL-C ratio	2.53 (2.40–2.66)	2.70 (2.58–2.82)	0.053
TG/HDL-C ratio	1.43 (1.30–1.56)	1.41 (1.29–1.53)	0.838
ApoAI (g/L)	1.52 (1.50–1.55)	1.48 (1.46–1.51)	0.024 *
ApoB100 (g/L)	1.04 (1.01–1.07)	1.08 (1.05–1.11)	0.036 *
ApoB100/ApoAI ratio	0.71 (0.68–0.73)	0.75 (0.73–0.78)	0.008 *

*: *p* < 0.05; LDL-C: low-density lipoprotein cholesterol; TG: triglycerides; HDL-C: high density lipoprotein cholesterol; ApoAI: apolipoprotein AI; ApoB100: apolipoprotein B100.

**Table 3:** Average cardiovascular risk compared between individuals with and without lactose intolerance genotype.

	rs4988235–T/T or T/C (Lactose Tolerance)	rs4988235–C/C (Lactose Intolerance)	*p*-Value
	Average Risk in % (95%CI)		
SCORE	2.23 (1.87–2.58)	2.95 (2.48–3.43)	0.039 *
SCORE2	5.49 (4.79–6.19)	7.09 (6.30–7.88)	0.001 *
PCE	5.35 (4.63–6.06)	6.76 (5.94–7.58)	0.005 *
RPCE	4.15 (3.54–4.76)	5.56 (4.74–6.39)	0.017 *
FRS–CVD	4.04 (3.41–4.68)	4.59 (3.97–5.22)	0.098
FRS–CHD	9.05 (8.04–10.07)	10.75 (9.52–11.97)	0.119

*: *p* < 0.05; SCORE: Systematic Coronary Risk Evaluation; SCORE2: Systematic Coronary Risk Evaluation 2; PCE: Pooled Cohort Equations; RPCE: Revised Pooled Cohort Equations; FRS–CVD: Framingham Risk Score for cardiovascular diseases; FRS–CHD: Framingham Risk Score for coronary heart disease.

## Data Availability

Due to data protection and ethical concerns, the dataset(s) supporting the conclusions of this article are available upon request from the study coordinators, Róza Ádány (adany.roza@med.unideb.hu) and Péter Pikó (piko.peter@med.unideb.hu).

## Supplementary Materials

The following supporting information can be downloaded at: https://www.mdpi.com/article/10.3390/nu17172741/s1, Table S1: Unadjusted descriptive comparisons between genotype groups in the Hungarian general population. Categorical variables were analyzed using Pearson’s chi-square test, and continuous variables using the Mann–Whitney U test. No covariate adjustment was applied in this table; Table S2: Unadjusted descriptive comparisons between genotype groups in the Roma population. Categorical variables were analyzed using Pearson’s chi-square test, and continuous variables using the Mann–Whitney U test. No covariate adjustment was applied in this table.

## Abbreviations

The following abbreviations are used in this manuscript:

ASCVD	atherosclerotic cardiovascular disease
CETP	cholesteryl ester transfer protein
CHD	coronary heart disease
CVD	cardiovascular disease
CVR	cardiovascular risk
FRS	Framingham Risk Score
GPMSSP	General Practitioners’ Morbidity Sentinel Stations Program
HDL	high-density lipoprotein
LCT	lactase
LDL	low-density lipoprotein
LNP	lactase non-persistence
LP	lactase persistence
MCM6	minichromosome maintenance complex component 6
mRNA	messenger RNA
PCE	Pooled Cohort Equations
RPCE	Revised Pooled Cohort Equations
SCORE	Systematic Coronary Risk Evaluation
SCORE2	Systematic Coronary Risk Evaluation 2
TC	total cholesterol
TG	triglycerides
