# Supplementary material for: Lactase Persistence-Associated rs4988235 Polymorphism: A Novel Genetic Link to Cardiovascular Risk via Modulation of ApoB100 and ApoAI
Source: Nutrients. 2025 Aug 24;17(17):2741. doi: 10.3390/nu17172741 (PMC12429906; doi:10.3390/nu17172741)
Supplement: Supplementary file 1 [file nutrients-17-02741-s001.zip › Supplementary Table S1.pdf]

Supplementary Table S1. Unadjusted descriptive comparisons between genotype groups in the Hungarian general population. Categorical variables were analyzed using Pearson's chi-square test, and continuous variables using the Mann–Whitney U test. No covariate adjustment was applied in this table.

		rs4988235 – T/T or T/C (lactose tolerance) N = 160	rs4988235 – C/C (lactose intolerance) N = 237	p-value
		Prevalence in % (95%CI)		
Women		51.48 (45.13 – 57.79)	61.25 (53.55 – 68.54)	0.055
Lipid lowering treatment		6.33 (3.75 – 9.97)	7.50 (4.17 – 12.35)	0.649
Antihypertensive treatment		27.85 (22.44 – 33.80)	29.38 (22.73 – 36.75)	0.741
Antidiabetic treatment		6.75 (4.07 – 10.48)	5.00 (2.39 – 9.21)	0.473
Current smoker		31.22 (25.58 – 37.32)	35.00 (27.93 – 42.61)	0.432
Lactose-free diet		2.53 (1.06 – 5.14)	2.50 (0.85 – 5.83)	0.984
Education	Primary	21.94 (17.03 – 27.53)	20.63 (14.92 – 27.39)	0.942
	Secondary	59.07 (52.73 – 65.19)	60.63 (52.92 – 67.95)	
	College or university	18.99 (14.39 – 24.34)	18.75 (13.29 – 25.33)	
rs1532624 in the *CETP* gene	C/C – genotype	27.43 (22.04 – 33.35)	34.38 (27.35 – 41.96)	0.270
	A/C – genotype	49.79 (43.46 – 56.12)	47.50 (39.87 – 55.22)	
	A/A – genotype	22.78 (17.80 – 28.43)	18.13 (12.75 – 24.64)	
rs5882 in the *CETP* gene	G/G – genotype	10.55 (7.12 – 14.93)	9.38 (5.58 – 14.62)	0.135
	G/A – genotype	48.95 (42.63 – 55.29)	40.00 (32.64 – 47.71)	
	A/A - genotype	40.51 (34.40 – 46.84)	50.63 (42.93 – 58.30)	
		Average (95%CI)		p-value
Age (years)		44.63 (43.08 – 46.18)	43.50 (41.56 – 45.44)	0.441
Waist circumference (cm)		95.81 (94.01 – 97.61)	96.36 (93.85 – 98.87)	0.861
BMI (kg/m^2)		27.35 (26.69 – 28.00)	27.17 (26.25 – 28.08)	0.702
Systolic blood pressure (mmHg)		126.71 (124.90 – 128.53)	126.81 (124.37 – 129.25)	0.977
Diastolic blood pressure (mmHg)		79.05 (77.92 – 80.18)	78.48 (77.08 – 79.88)	0.466
Insulin level (mU/L)		16.76 (14.38 – 19.14)	14.44 (12.00 – 16.88)	0.287
Fasting glucose (mmol/L)		5.36 (5.10 – 5.63)	5.09 (4.83 – 5.35)	0.032*
Uric acid (μmol/L)		294.97 (285.50 – 304.44)	276.68 (264.92 – 288.44)	0.016*
Creatinine (μmol/L)		67.00 (65.29 – 68.71)	63.97 (61.81 – 66.14)	0.021*
GGT (U/L)		34.81 (31.06 – 38.55)	33.55 (28.88 – 38.21)	0.407

CETP: Cholesteryl ester transfer protein gene; BMI: body-mass index; GGT: gamma-glutamyl transferase; A: adenine; C: cytosine; G: guanine; T: thymine; *: p <0.05
