# Supplementary material for: Lactase Persistence-Associated rs4988235 Polymorphism: A Novel Genetic Link to Cardiovascular Risk via Modulation of ApoB100 and ApoAI
Source: Nutrients. 2025 Aug 24;17(17):2741. doi: 10.3390/nu17172741 (PMC12429906; doi:10.3390/nu17172741)
Supplement: Supplementary file 1 [file nutrients-17-02741-s001.zip › Supplementary Table S2.pdf]

Supplementary Table S2. Unadjusted descriptive comparisons between genotype groups in the Roma population. Categorical variables were analyzed using Pearson's chi-square test, and continuous variables using the Mann–Whitney U test. No covariate adjustment was applied in this table.

		rs4988235 – T/T or T/C (lactose tolerance) N = 241	rs4988235 – C/C (lactose intolerance) N = 127	p-value
		Prevalence in % (95%CI)		
Women		68.50 (60.08 – 76.10)	76.35 (70.69 – 81.38)	0.104
Lipid lowering treatment		9.45 (5.27 – 15.45)	12.86 (9.09 – 17.53)	0.332
Antihypertensive treatment		30.71 (23.19 – 39.10)	31.95 (26.31 – 38.03)	0.807
Antidiabetic treatment		8.66 (4.69 – 14.49)	12.45 (8.73 – 17.06)	0.272
Current smoker		57.48 (48.79 – 65.83)	70.54 (64.56 – 76.02)	0.012
Lactose-free diet		---	0.83 (0.17 – 2.64)	---
Education	Primary	88.19 (81.73 – 92.93)	84.65 (79.69 – 88.77)	0.539
	Secondary	11.81 (7.07 – 18.27)	14.94 (10.87 – 19.85)	
	College or university	---	0.41 (0.04 – 1.92)	
rs1532624 in the *CETP* gene	C/C – genotype	37.01 (28.98 – 45.62)	32.37 (26.70 – 38.46)	0.355
	A/C – genotype	44.09 (35.67 – 52.78)	42.32 (36.21 – 48.62)	
	A/A – genotype	18.90 (12.83 – 26.36)	25.31 (20.14 – 31.07)	
rs5882 in the *CETP* gene	G/G – genotype	15.20 (9.73 – 22.26)	22.41 (17.49 – 27.98)	0.236
	G/A – genotype	47.20 (38.60 – 55.93)	41.08 (35.00 – 47.37)	
	A/A - genotype	37.60 (29.47 – 46.30)	36.51 (30.63 – 42.72)	
		Average (95%CI)		p-value
Age (years)		41.42 (39.27 – 43.57)	43.56 (42.02 – 45.10)	0.117
Waist circumference (cm)		96.28 (93.07 – 99.48)	93.99 (91.94 – 96.04)	0.286
BMI (kg/m^2)		28.19 (26.93 – 29.44)	27.30 (26.45 – 28.15)	0.298
Systolic blood pressure (mmHg)		122.08 (119.49 – 124.67)	124.56 (122.22 – 126.91)	0.453
Diastolic blood pressure (mmHg)		79.15 (77.61 – 80.69)	79.88 (78.52 – 81.24)	0.591
Insulin level (mU/L)		14.60 (12.34 – 16.87)	17.76 (14.80 – 20.72)	0.935
Fasting glucose (mmol/L)		5.03 (4.75 – 5.31)	5.14 (4.94 – 5.33)	0.198
Uric acid (µmol/L)		264.46 (252.03 – 276.90)	253.95 (244.42 – 263.47)	0.133
Creatinine (µmol/L)		63.79 (61.17 – 66.41)	61.34 (59.26 – 63.41)	0.073
GGT (U/L)		33.26 (27.62 – 38.90)	38.70 (26.81 – 50.59)	0.253

CETP: Cholesteryl ester transfer protein gene; BMI: body-mass index; GGT: gamma-glutamyl transferase; A: adenine; C: cytosine; G: guanine; T: thymine; *: $p < 0.05$
